# Computationally Efficient Wildfire Detection Method Using a Deep Convolutional Network Pruned via Fourier Analysis

**DOI:** 10.3390/s20102891

**Published:** 2020-05-20

**Authors:** Hongyi Pan, Diaa Badawi, Ahmet Enis Cetin

**Affiliations:** Department of Electrical and Computer Engineering, University of Illinois at Chicago, Chicago, IL 60607, USA; dbadaw2@uic.edu

**Keywords:** wildfire detection, block-based analysis, transfer learning, Fourier analysis, pruning and slimming

## Abstract

In this paper, we propose a deep convolutional neural network for camera based wildfire detection. We train the neural network via transfer learning and use window based analysis strategy to increase the fire detection rate. To achieve computational efficiency, we calculate frequency response of the kernels in convolutional and dense layers and eliminate those filters with low energy impulse response. Moreover, to reduce the storage for edge devices, we compare the convolutional kernels in Fourier domain and discard similar filters using the cosine similarity measure in the frequency domain. We test the performance of the neural network with a variety of wildfire video clips and the pruned system performs as good as the regular network in daytime wild fire detection, and it also works well on some night wild fire video clips.

## 1. Introduction

Early wildfire detection is of utmost importance to combat the unprecedented scale of wildfires happening all over the world. Recently, there has been a notable interest in developing real-time algorithms to detect wildfires using regular video-based surveillance systems [1,2,3,4,5,6,7,8,9,10,11,12,13,14,15,16,17,18,19,20]. Video-based forest fire detection can be used to replace traditional point-sensor type detectors because a single pan-tilt-zoom type camera can monitor a wide area, detect forest fire and smoke immediately after the start of the wildfire—as long as the smoke is within the viewing range of the camera. Nowadays, with the development of 5G communication [21,22], unmanned aerial vehicles (UAVs) have also become a good option for wildfire surveillance tasks because of their flexibility compared to fixed surveillance towers. However, all of the traditional video-based methods [1,2,3,4,5,6,7,8,9,10,11,12,13,14,15,16,17,18,19,20] rely on choosing features manually. In contrast, deep neural networks can extract relevant features by themselves given sufficient training data.

In recent years, deep learning has been widely used in a myriad of computer vision applications because of its high recognition capability. To the best of our knowledge, Gunay et al. [23] is the first paper to use deep learning in dynamic texture recognition including wildfires. In early deep learning based forest detection, researchers designed blank convolutional neural networks and trained them with collected or synthesized images [24,25,26,27,28]. For example, Zhao et al. designed a 15-layer convolutional neural network (CNN) to detect the forest fire [25]. What is more, to locate the fire and smoke in frames, Barmpoutis et al. [29], Huang et al. [30] and Chaoxia et al. [31] proposed R-CNN -based fire detection method. However, neural network training requires huge amounts of data, while such data can be very expensive and infeasible. If the training data is insufficient, the network may not be robust. To overcome this problem, some researchers adopted transfer learning [32] to design neural networks. Transfer learning is a very efficient method and it is widely used in recognition tasks because of its advantage that we only need to train only the final several layers instead of the whole network. Transfer learning requires a smaller dataset for training and can avoid overfitting. Typical examples are AlexNet-based CNN by Muhammad et al. [33] and YOLO -based CNN by Park et al. [34] and Jiao et al. [35]. Inspired by them, in this work, we propose to use transfer learning from MobileNet-V2 [36] for forest fire detection. Moreover, after updating the network with the available data, we notice that some kernels (filters) have very low energy. Computing these low-energy kernels is a waste of resources since their output is insignificant compared with the outputs of other kernels. We also observe that some kernels have very similar frequency responses, which means storing only one from each pair of similar kernels can significantly reduce storage memory for edge devices. We take advantage of these two facts and prune the kernels according to their frequency response via Fourier analysis. Fourier analysis not only trims the deep neural network but also removes the unnecessary convolutional units. The trimmed network provides as good results as the regular network. Fourier domain trimming is not specific to the wildfire detection task, it can be used in trimming other networks as well. We take advantage of these two facts of low-energy and similar kernels and prune the kernels according to their frequency response via Fourier analysis.

Compared with other deep learning methods [23,24,25,26,27,28,29,30,31,33,34,35], the major advantage is that, after transfer learning, we prune the network via Fourier analysis. More specifically, we have the following advantages:

- Our method takes advantage of MobileNet-V2 [36] in efficiency. Details of this aspect are provided in Section 3.
- We prune and slim the convolutional and dense layers according to frequency response of kernels using Fourier analysis in order to accelerate the inference of the neural network and save storage. Details of this aspect are provided in Section 3.1 and Section 3.2.
- We detect wildfire in overlapping windows so we can easily detect smoke even if it exists near the edge of a frame. We achieve this by dividing the frame in many blocks and detecting the smoke block-by-block. Details of this aspect are provided in Section 4.
- Compared to R-CNN [30,31], and YOLO method [34,35], our block-based analysis makes the building of testing and training datasets easy because we mark only the blocks containing fire. We only need to label each block as fire or no-fire instead of marking the region of fire and smoke in a given frame using several bounding boxes. Making and updating the dataset in our method is much easier compared to R-CNN and YOLO method. In wildfire surveillance task, knowing the fire in which image block is sufficient for fire departments to take action. In addition, compared to frame-based methods, block-based analysis allows us to determine capture very small fire regions and smoke.
- Our system can detect very small smoke regions, while papers from related works [24,25,26,27,28,29,30,31] have not provided the test results on such small smoke regions. The details of this aspect are provided in Section 5.2.
- The input of our system is in 1080P and it can also be adjusted for higher resolution. Thus, our method matches common surveillance cameras, since down-sampling always causes information loss and may make small regions of smoke invisible. According to our experimental results, our method works well even if the smoke region is very small.
- After testing the performance on daytime surveillance and obtaining a very good result, we further tested our system system with night events, and it works on many video clips.

## 2. Dataset for Training

**Figure 1:**
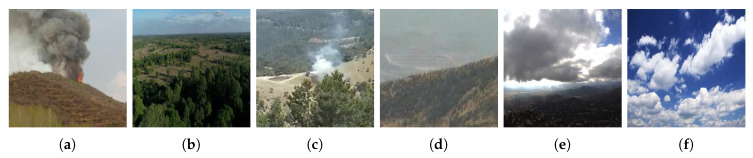
Samples of our training dataset. (**a**,**b**) are gathered from the Internet; (**c**,**d**) are from FIRESENSE Database; (**e**,**f**) are cloud images.

**Figure 2:**
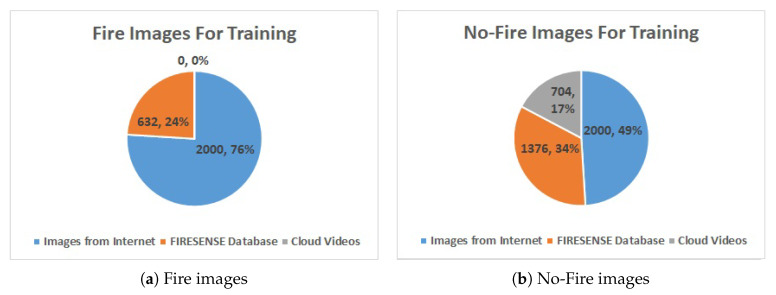
Image distribution of training dataset.

We use the dataset of Reference [28] which contains 4000 images gathered from the Internet (half with fire and half without fire) shown in Figure 1a,b, and FIRESENSE database [37] shown in Figure 1c,d which contains 11 fire videos and 16 no-fire videos. During practice, we notice that the system would false alarm at cloud region sometimes, so we also add 4 cloud video clips shown in Figure 1e,f into our training dataset to reduce the false-alarm rate. Figure 2 shows the distribution of our training dataset.

In order to avoid the over-fitting during training, we only keep the first frame in each 20–100 frames (depends on the length of the video) to maintain diversity because the Internet images are in variety of scenarios compare to the video clips. If we train too much on the video clips, the neural network may easily over-fit on the scenes in these video clips. All images and frames are resized into 224×224 as the input of the neural network, and 5% of them are picked randomly for validation. Data augmentation by shifting and translating wildfire images with filling by reflect translation as in Reference [28] is also adopted here. Let $Y\in R^{M\times N}$ denote the original image and index start from 0, if we want to shift the image by *m* length up and *n* length left (negative value means shifting in the opposite direction), then the augmented image $\tilde{Y}$ can be represent as

(1) $$\begin{array}{ccc} \; & {\tilde{Y}}_{i,j}=\; & \left\{\begin{array}{cc} Y_{\min(i+m,2M-i-m-2),\min(j+n,2N-j-n-2)} & m\geq 0,n\geq 0\\ Y_{\left|i+m|,\min(j+n,2N-j-n\right)} & m<0,n\geq 0\\ Y_{\min(i+m,2M-i-m-2),|j+n|} & m\geq 0,n<0\\ Y_{\left|i+m|,|j+n\right|} & m<0,n<0, \end{array}\right. \end{array}$$

where, i=0,1,…,M−1, j=0,1,…,N−1. Range of *m* and *n* depends on the location of smoke in each image and frame. The reason for using such an augmentation scheme is to ensure that the classifier will see examples in which wildfire starts from different locations during training.

## 3. Fourier Transform Based Pruning the Network

We use the MobileNet-V2 [36] for wildfire recognition after retraining. MobileNet-V2 is an efficient convolutional neural network for mobile vision applications. MobileNet’s energy consumption is much smaller than other neural networks. Usually, wildfire monitoring cameras are placed in remote locations and/or UAVs. In such cases, energy consumption is very important. Therefore, MobileNet-V2 is suitable for wildfire detection. We replace the dense layers of MobileNet-V2 by a new dense layer with two neurons, and we apply softmax function as the activation function in the dense layer for decision making. The existence of fire is labeled as 1 and “no-fire” is labeled as 0. The probability *P* of fire can be represent as

(2) $$P=\frac{\exp(P_{1})}{\exp\left(P_{1}\right)+\exp\left(P_{0}\right)},$$

where $P_{0}$ and $P_{1}$ represent the outputs of the two neurons before activating, respectively.

In this paper, we employ TensorFlow in Python 3 to train the network and then, convert the well-trained model to a TensorFlow lite model. We can install this model on a NVIDIA Jetson Nano board or a similar board.

In the following section we will describe how we can further trim the network.

### 3.1. Pruning Low-Energy Kernels

A well-trained CNN may contain some redundant neurons—no matter what the input is, the output of these neurons will always take small values. Such neurons have filter weights with too small values. As we all know, performing a convolution operation is relatively expensive, especially in a long-time surveillance task. Thus, efficiency can be significantly improved if we can avoid calculation with too small weights.

**Figure 3:**
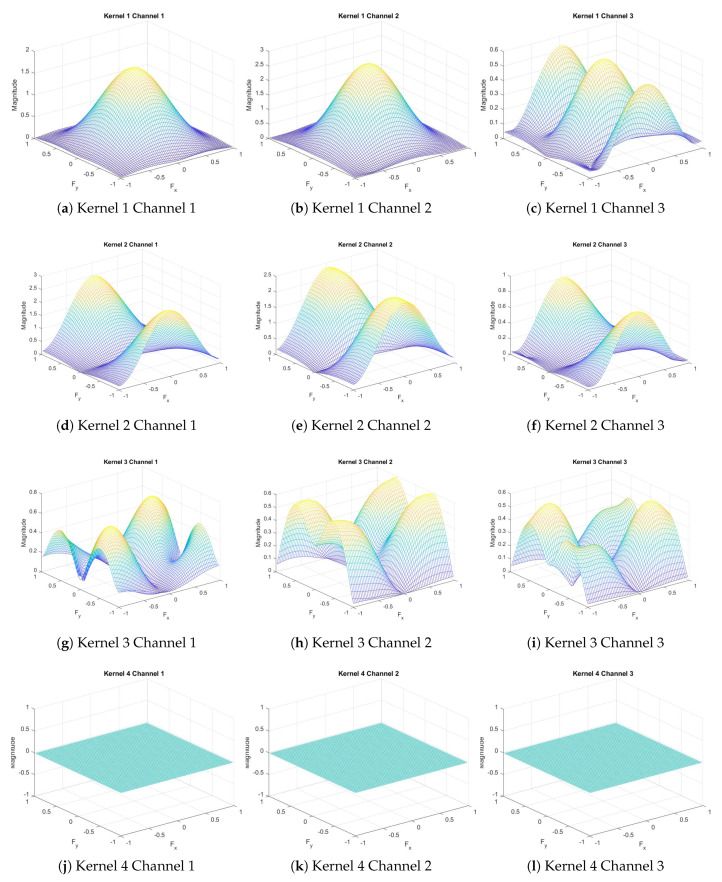
Frequency response of the first convolutional layer.

**Figure 4:**
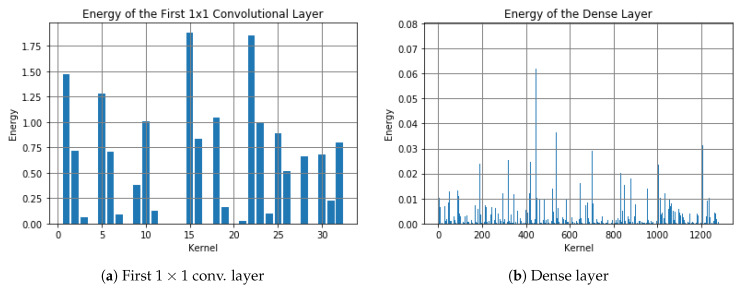
Energy distribution.

**Figure 5:**
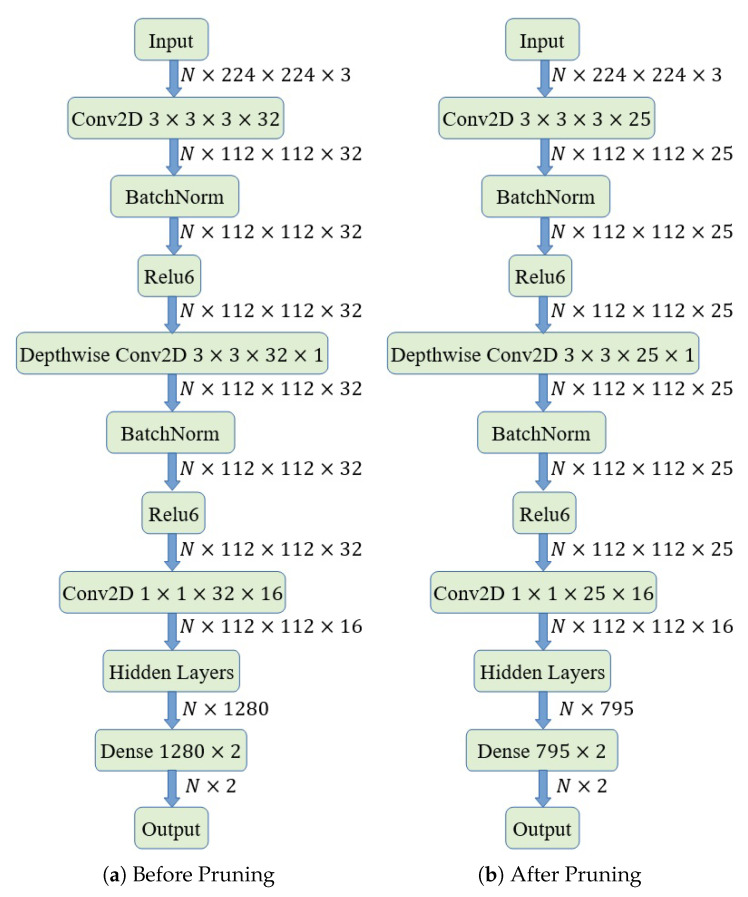
Pruning graph, “*N*” is batch size.

The MobileNet-V2 model we fine-tuned has 18 3×3 convolutional layers. When we extract weights from each 3×3 layer and calculate their DFT via MATLAB, we find that there are some with almost zero-magnitude response only in the first standard convolutional layer. It contains 32 3-channel 3×3 kernels, and some of their frequency responses are shown in Figure 3. We find that the magnitude responses of kernels with numbers 4, 8, 12–14, 20, 29 are close to 0 for all frequency values. Then, we calculate the energy of the first projection layer among the input direction as shown in Figure 4a, we notice that the energy with these indexes is also close to 0. After pruning these layers by removing those weights (the first standard convolutional layer is 3×3×3×32, the first depthwise separable convolutional layer is 3×3×32×1, and the projection convolutional layer is 1×1×32×16 before pruning, then each “32” reduces to “25”), we save 21.875% computation in these layers. Moreover, when we plot the energy distribution of the dense layer as shown in Figure 4b, we find that there are many weights having low energy. By removing these kernels in the dense layer with energy less than 0.0005 and corresponding kernels in the previous layers, we save 485/1280=37.89% computation in these layers. Figure 5 shows the graph before and after pruning. By running the inference 100 times and calculating the average time consumption on the computer, we find that it takes 0.499921 s before pruning, and takes 0.464778 s after pruning. Thus, we totally save 7.04% time by pruning these layers.

### 3.2. Slimming Similar Kernel Pairs

**Figure 6:**
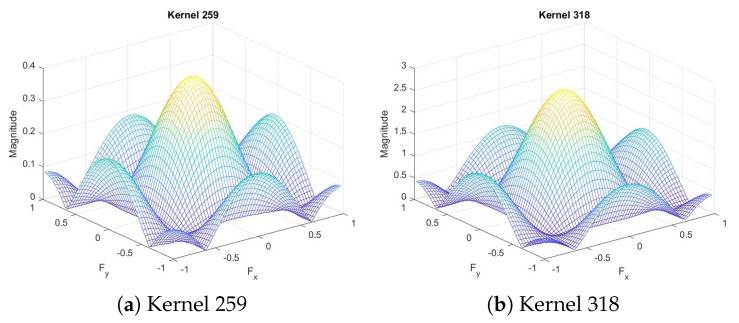
Frequency response of the final convolutional layer.

In other depthwise separable convolutional layers, on the other hand, when we plot the magnitude, we notice that some have similar shapes. For example, in the final convolutional layer, we notice that the magnitudes of kernel 259 and kernel 318, shown in Figure 6, are very similar in shape, though different in scale, which means we can slim the convolutional layer by saving only one of each similar kernel pairs and reconstruct the other one by scaling the output of the first one.

We compute the frequency response of the filters of the network and keep only one of the filters with similar magnitude frequency responses. We also discard filters with magnitude responses significantly smaller than the other filters. More specifically, we calculate cosine similarity of each kernel pair:

(3) $$\mathrm{Filter}\;\mathrm{similarity}=\cos\left(\theta \right)=\frac{<X,Y>}{∥X∥\cdot ∥Y∥},$$

where X and Y are the Fourier transform magnitudes of the two filters in vector form, respectively. Since we use the magnitudes of the frequency responses, the filter similarity measure is in the range of [0,1]. We treat the kernels with similarity larger than 0.99925 as a pair of similar kernels and store only one of them. We cannot compare the filters in spatial domain using their weights because there may be a phase shift between the two filters and the filters may look different but they can essentially be the same. Cosine similarity measure also handles the case of the two filters with

(4) $$|Y\left[k_{1},k_{2}\right]|\approx \alpha |X\left[k_{1},k_{2}\right]|,$$

where *X* and *Y* are the DFT or the two kernels, respectively. Both of these filters will generate very similar outputs for a given input except that one of the outputs will be a shifted and scaled version of the other output.

Table 1 has the summary of our results of pruning and slimming. The first one is a standard layer named “conv” and others are depthwise separable named “expanded_conv”, and between each depthwise separable convolution layer, there are two 1×1 standard convolutional layers to change the dimensions. In fact, this idea should be able to apply to all MobileNet-V2 based neural networks, because we find that the original MobileNet-V2 also contains both very low-energy kernels and similar kernels.

**Figure 7:**
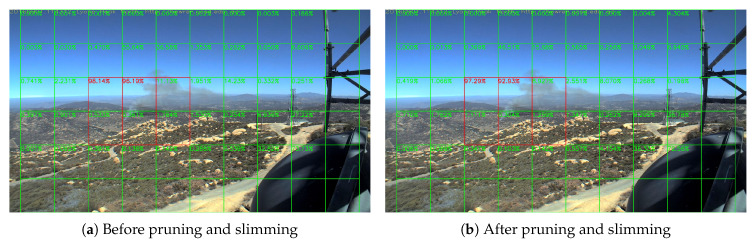
Lyons Fire in San Diego, 1 September 2016.

Figure 7 shows the output of a smoke frame before and after pruning and slimming. More statistics are provided in Section 5.2 and Section 5.4. We can see that the result changes negligibly. In this way, although these layers cannot be accelerated even with similar kernels because the input of each kernel is still different, we save 22.59% storage of the weights, which is very important to edge devices.

## 4. Block-Based Analysis of Image Frames

Nowadays, with the development of technology, we can obtain forestry surveillance videos in 1080P or higher resolution. However, if we design the input of the neural network in these high resolutions, the network will be too huge to train. Most common method to use to solve this problem is to down-sample the images, which correspondingly will increase the difficulty to detect little smoke plumes and will cause information loss. To overcome this problem, we divide the image frames into small blocks as in [28]. Suppose that the width and height of the effective image region is $M_{i}$ and $N_{i}$, and the width and height of each block is $M_{b}$ and $N_{b}$, then the row number *R* and the column number *C* of each block can be represent as

(5) $$R=\left\lfloor \frac{H_{i}}{H_{b}}\right\rfloor ,C=\left\lfloor \frac{W_{i}}{W_{b}}\right\rfloor ,$$

where ⌊⌋ is the floor function. We divide the frames into many tiles as in Figure 7. The videos are in 1080 P (HPWREN videos are in 6 MP in fact, so we down-sample them into 1080 P for convenience. In real-world applications, we can also use different division strategy to make good use of higher resolution), and then divide them into many tiles with 180×180 pixels, and every 2 by 2 tiles consist a block with 360×360 pixels. The score is the fire rate of each block, composed of the tile has score and its bottom, right and bottom-right tiles, rather than the forest fire rate of each tile. This is the reason that there is no score in the most right tiles and the most bottom tiles. In this way, a frame is divided into 5×9=45 blocks, and if the smoke exists at the edge of one block, it will also exist at the center of its neighbor block. Then, we resize each block into the input size and then feed the network with resized blocks. Therefore, the input of the neural network is 45×224×224×3.

## 5. Network Performance

In this section, we will test the speed and accuracy of our model with the HPWREN dataset [38] and some YouTube forest fire videos. Unlike other binary classification tasks (like the famous Kaggle’s competition, cats VS dogs), forest fire detection requires a very low false-alarm rate, otherwise the system will produce false alarming in the long-term surveillance task. This may bother the security guards verifying the system outputs. If there are too many false alarms they may develop the bad habit of ignoring the wildfire monitoring system. To reduce the false-alarm rate, we set the alarm threshold value as 0.95, such that the system will alarm only if the score is large than this value. In practical the real-world applications, we may decrease the threshold value to 0.85, 0.9 or other values to increase the fire detection rate, if no dust, cloud or other confusing objects exist in the monitored area, or increase if they exist too much.

### 5.1. Speed Test

In this section, we test the speed of our model on the NVIDIA Jetson Nano board. We are only interested in the total time of each frame to finish frame pre-processing (division and resizing), and interpretation of the neural network, because time consumed by signal transmission depends on the surveillance cameras and the network and should be finished in an instant. Our system takes about 3 to 4 s to process a single frame. This delay is negligible compare to the time that fire departments take to take actions.

### 5.2. Daytime Fire Surveillance Test

**Figure 8:**
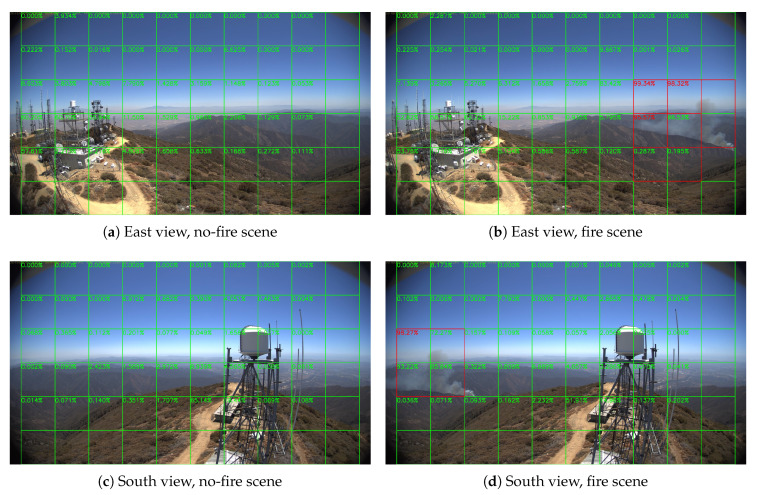
Holy Fire around Santiago Peak, 6 August 2018.

**Figure 9:**
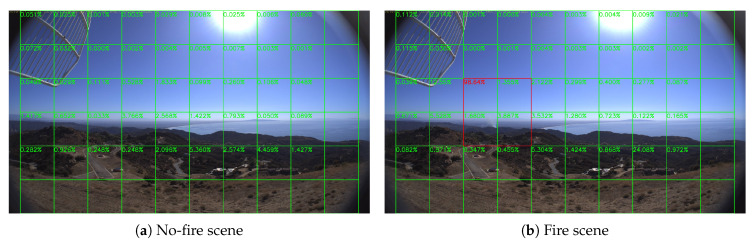
Palisades fire, 21 October 2019.

**Figure 10:**
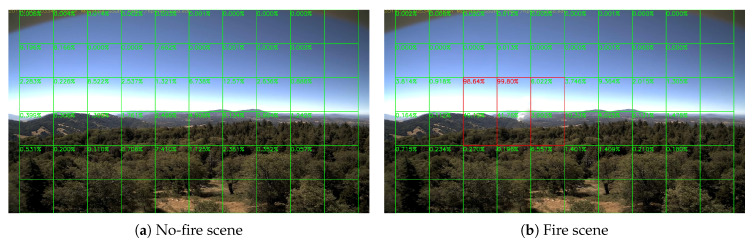
Banner fire, 3 June 2014.

**Figure 11:**
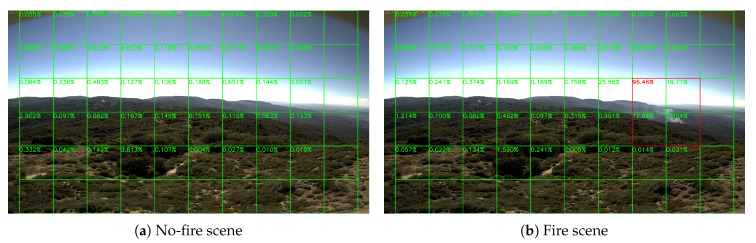
Palomar Mountain fire, 24 July 2015.

**Figure 12:**
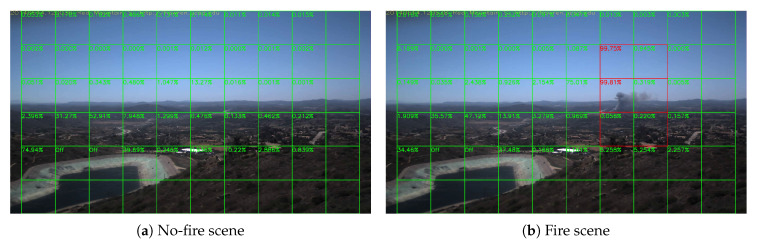
Highway fire, 14 May 2014.

**Figure 13:**
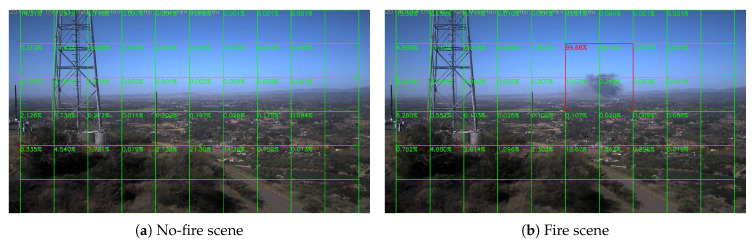
Tomahawk fire, 14 May 2014.

**Figure 14:**
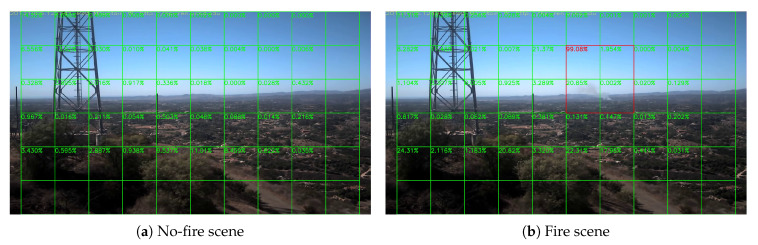
DeLuz fire, 5 October 2013.

Sample results of daytime fire surveillance test are shown in Figure 7, Figure 8, Figure 9, Figure 10, Figure 11, Figure 12, Figure 13 and Figure 14, and Table 2 lists the frame number that fire starts in each video clips and the first frame number that our system manages to detect the fire. We also provide the result before pruning and slimming in Table 2. Only two videos a have one or two frame detection delay after pruning and slimming. It is worth pointing out that although smoke regions in Figure 9, Figure 10 and Figure 11 and Figure 14 are very small, our system still works. According to Table 2, our system can detect smoke timely after fire occurs. Moreover, as shown in Table 3, our system has been tested with some YouTube daytime fire videos. All fire events in these videos have been detected successfully. However, fire occurs at the beginning of these videos and many are already large, so we only record the name and resolution of these videos.

### 5.3. Night Fire Surveillance Test

**Figure 15:**
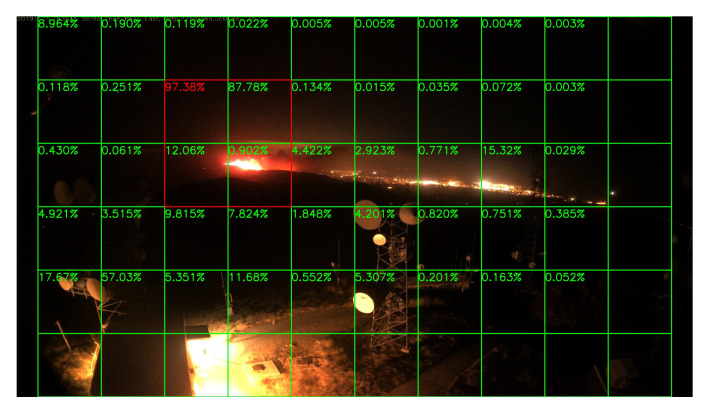
CaveFire, 25 November 2019.

**Figure 16:**
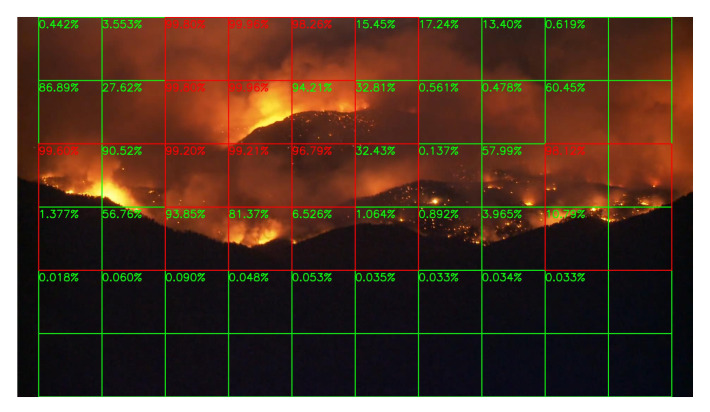
Boulder Colorado Fire, 6 September 2010.

Although there are only a few images in night scenes in our training dataset, it is still worthy to test the network’s performance at night. Unfortunately, compare to the daytime surveillance, smoke during the night is almost invisible, and the fire is very similar to the city lights like Figure 15, unless the fire is already very large like Figure 16. Thus, night fire detection under a color camera is very challenging, and performance will be better if infrared cameras are used instead of color cameras, but unluckily, there are few infrared video clips or images about night fire available on the Internet. Without sufficient data, it is difficult to train the network well.

**Figure 17:**
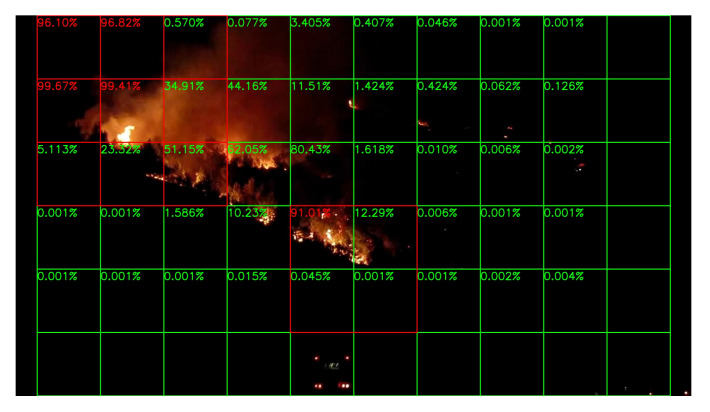
Night forest fire, 28 August 2011.

**Figure 18:**
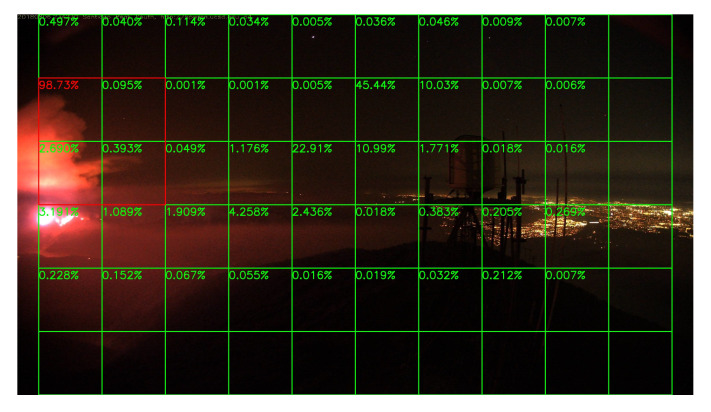
Holy Fire, 6 August 2018.

Sample results of night fire detection are shown in Figure 15, Figure 16, Figure 17 and Figure 18. Unlike the daytime surveillance, there are very few night fires, so we cannot conduct a detection table similar to Table 2 which lists the initial frame number when the fire starts in this section. Our system still detects all the night fire events in some videos.

### 5.4. Performance on No-Fire Videos

**Figure 19:**
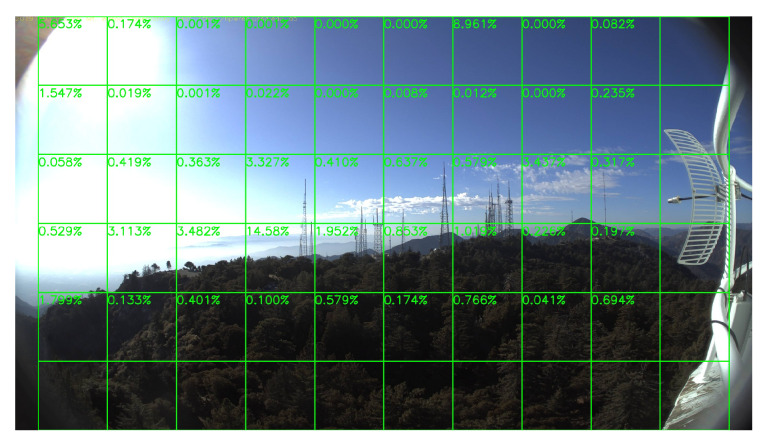
A HPWREN no-fire daytime frame, 3 November 2019.

**Figure 20:**
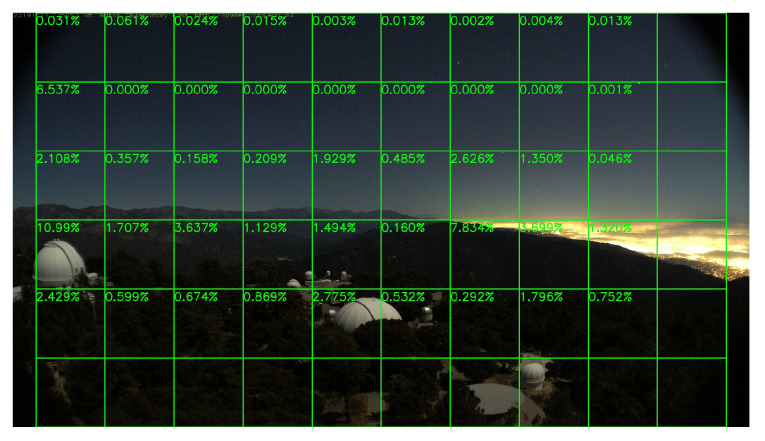
A HPWREN no-fire night frame, 11 November 2019.

In this section, we run our model on a group of long video clips to test our false-alarm rate, and the result is shown in Table 4. All videos record by whole days, which means they contain both daytime and night scenes. Figure 19 and Figure 20 show a daytime no-fire scene and a night no-fire scene respectively. We can see that the false-alarm rates on all video clips are less than 0.22% except only one video clip with an unexpected light with a long duration at night, and more than half of them are less than 0.1%. Thus, our false-alarm rate is satisfying for a real-world forest fire surveillance task.

**Figure 21:**
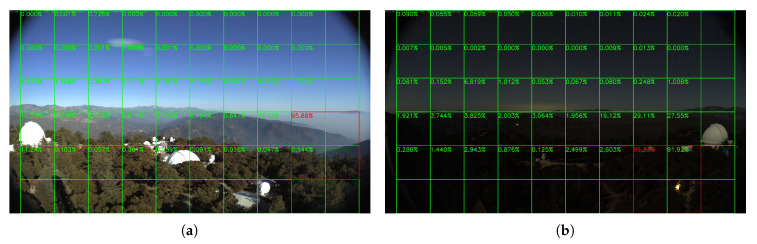
False Alarms. (**a**) False alarm because of dust, 3 November 2019. (**b**) False alarm because of light, 10 November 2019.

Now we will explain some false-alarm scenarios. As shown in Figure 21a, a system releases false alarms because of dust. If it is omitted, we may also miss some small smoke. On the other hand, this kind of false alarm is in single frames and then disappears for a few more frames, so it will not cause too much trouble. Moreover, this kind of false alarm can be overcome by increasing the alarm threshold value as we mentioned in the first paragraph of Section 5.

Another scenario where our system releases false alarms is because of light like in Figure 21b. Unlike a large region of light like in Figure 15, light only existing in a very small region looks like a spark. If it is omitted, we may also miss the real spark. Moreover, in a fixed surveillance tower, we can manually set the system to ignore this block during the time the light is on.

### 5.5. Comparison with Other Methods

**Figure 22:**
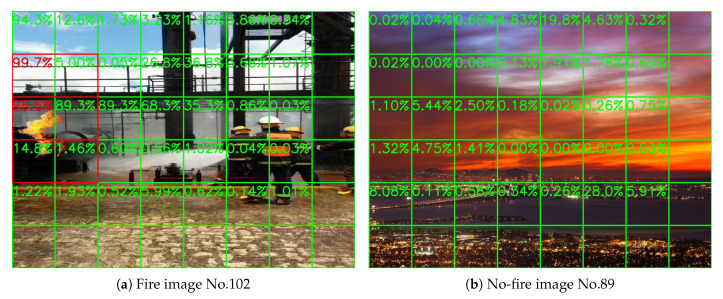
Test result on the BoWFire dataset.

In this section, we will compare our method with some other related works. It is hard to compare the results from the HPWREN dataset with other methods because most of them are not open source, nor state their training setting. Here we use the BoWFire dataset [39] for comparison. It contains 119 fire images and 107 no-fire images, and it has been used as the test dataset by many related works [26,27,28,29,30,31]. The resolution of images in the BoWFire dataset are varied from 800×450 to 1365×1024, and many of them are portrait. So if we resize them into 1080 P, the images will be blurred and the portrait images will be deformed. So in this section, we resized them into 800×600 instead, or 600×800 if the image is portrait. Then we divide the frame into 5×7 or 7×5 blocks with block size is 200×200. Figure 22 shows two test result images, and Table 5 states our comparison with other methods. According to the table, our method and that of Reference [31] are tied for the highest accuracy, while our method works better with the lowest false-alarm rate.

Overall, in Section 5, we provided the test results on the daytime and night surveillance tasks. As shown in Figure 9, Figure 10 and Figure 11 and Figure 14, our system canbstill capture those very small smoke regions. According to Table 2, our system can detect smoke soon after a fire occurs. We also provided the false-alarm information for many long videos in Section 5.4. It shows that our system is very reliable in long-term surveillance task. At the end, we also observed two false-alarm cases in Section 5.4 with the analyzed reason why they happened and solutions that to avoid them by changing the threshold from 0.95 to a higher value, or manually blocking the sky and other possible confusing regions, if the system is installed in a fixed surveillance tower. At the end, we compared our method with some related works about the performance on the BoWFire dataset. Our method has the advantage of the lowest false-alarm rate. As we have mentioned in the first paragraph of Section 5, a very low false-alarm rate is very important to a long-term surveillance system.

## 6. Conclusions

In this paper, we proposed a block-based forest fire detection method via transfer learning. We introduced a novel pruning and slimming method using Fourier analysis to meet the lower time and storage requirements of edge devices. We use a block based strategy so it matches the high resolution of common surveillance cameras without performing any down-sampling. Down-sampling may cause information loss and small smoke plumes may become invisible. Therefore, our block based approach does not suffer from down-sampling and it can help locate the smoke locations as shown in Figure 7 We have tested various video clips in both daytime and night scenarios and obtained a satisfactory recognition performance. The system did not miss any smoke videos. The algorithm may not detect smoke in some frames but it detected all smoke events. This is because when the smoke is very large, its shape will be similar to the cloud, while we have added many cloud features in the training dataset to reduce false-alarms due to clouds. On the other hand, a well-developed forest fire surveillance system should be able to detect the fire immediately whenever it occurs within the viewing range of the camera. This can be achieved by fusing classical computer vision based wildfire detection methods and deep learning based methods.

In future work, we will add more images to our training dataset, which will improve the accuracy of our system. We will get more surveillance video clips from our installed wildfire monitoring cameras and new video clips from HPWREN. We will update our real-time forest fire surveillance system with the software described in this paper. Our NVIDIA Jatson Nano implementation was installed in one location and we will collect more data during the summer of 2020 and improve the proposed algorithm.

## Figures and Tables

**Figure 23:**
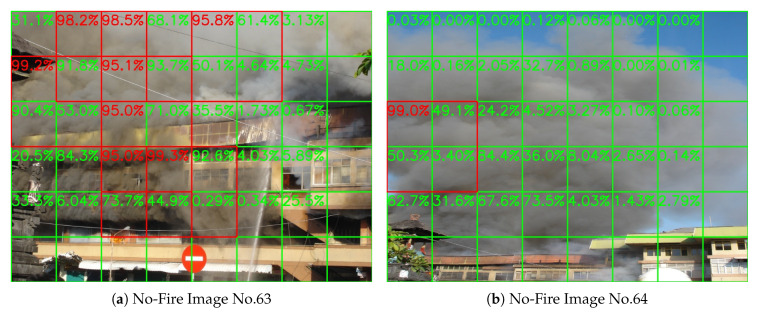
Two smoke images on the BoWFire no-fire test dataset.

**Table 1:** Pruning and slimming information of our model.

Layers Name	Kernels Num	Slimmed Num	Rate (%)
conv ${}^{a}$	32	7	21.88
expanded_conv ${}^{a}$	32	7	21.88
expanded_conv_1	96	2	2.08
expanded_conv_2 ${}^{b}$	144	0	0.00
expanded_conv_3	144	74	51.39
expanded_conv_4	192	6	3.13
expanded_conv_5 ${}^{b}$	192	0	0.00
expanded_conv_6	192	112	58.33
expanded_conv_7	384	9	2.34
expanded_conv_8	384	2	0.52
expanded_conv_9	384	3	0.78
expanded_conv_10	384	17	4.43
expanded_conv_11	576	2	0.35
expanded_conv_12	576	1	0.17
expanded_conv_13	576	490	85.07
expanded_conv_14	960	17	1.77
expanded_conv_15	960	45	4.69
expanded_conv_16	960	825	85.94
Slimming Overall ${}^{c}$	7104	1605	22.59

Expanded_conv is the name of depthwise separable convolutional layers. ${}^{a}$ These layers are pruned by removing weights with too low energy. ${}^{b}$ These layers cannot be slimmed even with lower threshold (0.9990), while too low threshold may cause the loss of accuracy. ${}^{c}$ Layers with ${}^{a}$ are excluded because they are using pruning method.

**Table 2:** Daytime Fire Result of HPWERN Database.

Videos Name	Resolution	Fire Starts	First Detected ${}^{a}$
Lyons Fire	1600×1200	156	164 (164)
Holy Fire East View	3072×2048	721	732 (732)
Holy Fire South View	3072×2048	715	725 (724)
Palisades Fire	3072×2048	636	639 (639)
Banner Fire	1440×1080	15	17 (17)
Palomar Mountain Fire	1440×1080	262	277 (275)
Highway Fire	1600×1200	4	6 (6)
Tomahawk Fire	1600×1200	32	37 (37)
DeLuz Fire	1440×1080	37	48 (48)

Fire appears some time after these videos start. Results in brackets in ${}^{a}$ are the result before pruning and slimming.

**Table 3:** Worked YouTube daytime fire video list.

Videos Name	Resolution
Barn Fire Overhaul in Marion County Oregon	2560×1440
Prairie Fire	1920×1080
Drone footage of DJI Mavic Pro Home Fire	1920×1080
Cwmcarn Forest Fire	3840×2160
Drone Footage of Kirindy Forest Fire	3840×2160
Drone Over Wild Fire	1920×1080
Fire in Bell Canyon	1920×1080
Forest Fire at the Grand Canyon	3840×2160
Forest Fire Puerto Montt by Drone	1920×1080
Forest Fire with Drone Support	1920×1080
Kirindy Forest Fire	3840×2160
Lynn Woods Reservation Fire	3840×2160
Prescribed Fire from Above	1920×1080
Semi Full of Hay on Fire I-70 Mile 242 KS Drone	1920×1080
Chimney Tops Fire	1920×1080

Fire occurs at the beginning of these videos and many are already large.

**Table 4:** No-Fire Video Result of HPWREN Database.

Videos Name	Frames Num	False-Alarm Num	False-Alarm Rate (%)
wilson-w-mobo-c	10,080	2	0.01984
wilson-s-mobo-c	10,074	2	0.01985
wilson-n-mobo-c	10,024	3	0.02993
wilson-e-mobo-c	10,028	43 ${}^{a}$	0.4288
vo-w-mobo-c	10,009	5	0.04996
69bravo-e-mobo-c	1432	1	0.06983
69bravo-e-mobo-c	1432	0	0.0000
syp-e-mobo-c	1421	3	0.2111
sp-n-mobo-c	1252	2	0.1597
sp-w-mobo-c	1282	1	0.07800
sp-s-mobo-c	1272	2	0.1572
sp-e-mobo-c	1278	2	0.1565

${}^{a}$ There is an unexpected long light shown in Figure 21b. We get same false-alarm result before and after pruning and slimming in threshold of 0.99925. With lower the slimming threshold (0.9990), the false-alarm rate will increase.

**Table 5:** Comparison with Other Methods.

Method	Detection Rate (%)	False-Alarm Rate (%)	Accuracy (%)
Muhammad et al. [26]	97.48	18.69	89.82
Muhammad et al. [33]	93.28	9.34	92.04
Chaoxia et al. [31]	92.44	5.61	93.36
Our Method	91.60	4.67 ${}^{a}$	93.36

${}^{a}$ There are two smoke images labeled as no-fire as shown in Figure 23. Our method managed to detect them, but we still count them as false-alarm cases here for comparison because they are not discussed in References [26,27,28,29,30,31]. If we count them as true-detected cases, then our three rates are 91.74%, 2.80% and 94.25%, respectively.

## Abbreviations

The following abbreviations are used in this manuscript:

UAV	Unmanned Aerial Vehicle
CNN	Convolutional Neural Network
DFT	Discrete Fourier transform
MP	Million Pixels
